# Detection of multiple strains of *Mycobacterium tuberculosis *using MIRU-VNTR in patients with pulmonary tuberculosis in Kampala, Uganda

**DOI:** 10.1186/1471-2334-10-349

**Published:** 2010-12-10

**Authors:** Katherine R Dickman, Lydia Nabyonga, David P Kateete, Fred A Katabazi, Benon B Asiimwe, Harriet K Mayanja, Alphonse Okwera, Christopher Whalen, Moses L Joloba

**Affiliations:** 1 Department of Pediatrics, Boston Medical Center, Boston University, Boston, USA; 2 Department of Medical Microbiology, Makerere University College of Health Sciences, Kampala, Uganda; 3 Department of Medicine, Makerere University College of Health Sciences, Kampala, Uganda; 4 Tuberculosis Unit, Mulago National Referral and Teaching Hospital, Kampala, Uganda; 5 Epidemiology and Biostatistics, College of Public Health, University of Georgia, Athens, USA

## Abstract

**Background:**

Many studies using DNA fingerprinting to differentiate *Mycobacterium tuberculosis *(MTB) strains reveal single strains in cultures, suggesting that most disease is caused by infection with a single strain. However, recent studies using molecular epidemiological tools that amplify multiple targets have demonstrated simultaneous infection with multiple strains of MTB. We aimed to determine the prevalence of MTB multiple strain infections in Kampala, and the impact of these infections on clinical presentation of tuberculosis (TB) and response to treatment.

**Methods:**

A total of 113 consecutive smear and culture positive patients who previously enrolled in a house-hold contact study were included in this study. To determine whether infection with multiple MTB strains has a clinical impact on the initial presentation of patients, retrospective patient data (baseline clinical, radiological and drug susceptibility profiles) was obtained. To determine presence of infections with multiple MTB strains, MIRU-VNTR (Mycobacterial Interspersed Repetitive Unit-Variable-Number Tandem Repeats) -PCR was performed on genomic DNA extracted from MTB cultures of smear positive sputum samples at baseline, second and fifth months.

**Results:**

Of 113 patients, eight (7.1%) had infection with multiple MTB strains, coupled with a high rate of HIV infection (37.5% versus 12.6%, *p *= 0.049). The remaining patients (105) were infected with single MTB strains. The proportions of patients with MTB smear positive cultures after two and five months of treatment were similar. There was no difference between the two groups for other variables.

**Conclusion:**

Infection with multiple MTB strains occurs among patients with first episode of pulmonary tuberculosis in Kampala, in a setting with high TB incidence. Infection with multiple MTB strains had little impact on the clinical course for individual patients. This is the first MIRU-VNTR-based study from in an East African country.

## Background

*Mycobacterium tuberculosis *(MTB) is among the most successful human pathogens worldwide and is responsible for extensive morbidity and mortality, with approximately 2 million deaths each year thought to be due to primary infection, endogenous reactivation of primary infection, or exogenous re-infection with a new strain [1]. Molecular epidemiological studies on transmission dynamics [2,3] as well as studies on recurrent tuberculosis (TB) [4,5] have differentiated the MTB complex strains causing disease. Most studies using DNA fingerprinting to differentiate mycobacterial strains have shown single strains in cultures, suggesting that most disease is caused by infection with a single strain [6]. However, recent studies using PCR (Polymerase Chain Reaction) -based molecular epidemiological tools that amplify multiple targets have demonstrated simultaneous infection with multiple strains of MTB. Different strains have been found in the same sputum sample [7-11] or different sputum samples from the same patient [7-9], and in different diseased anatomical sites of the patient [12,13].

The prevalence of infections with multiple MTB strains in a population has implications for the interpretation of molecular typing methods, as well as newer molecular methods for detecting drug resistance. Infection with multiple strains of MTB may be erroneously classified as exogenous re-infection or laboratory cross-contamination. Patients may be infected with both drug resistant and susceptible strains, of which only one is detectable through drug resistance testing. This could affect decisions regarding tuberculosis control and predictions for patients' clinical responses to therapy. The ability of MTB strains to infect a host without generating immunity to infection by other strains also has implications for vaccine development. We are yet to fully understand the prevalence, implication, and factors associated with infections with multiple MTB strains for virulence, response to therapy, and clinical presentation.

This study aimed at determining the prevalence of infection with multiple MTB strains in a high TB incidence setting in Kampala (Kawempe division), Uganda, using "Mycobacterial Interspersed Repetitive Unit (MIRU)-Variable-Number Tandem Repeats (VNTR)" analysis. The incidence of TB in Kawempe division is high at 920 per 100,000 population per year, similar to many urban areas in Sub-Saharan Africa [14,15]. Additionally, because no studies have followed patients with multiple infections through their treatment course, we aimed to determine whether infection with multiple MTB strains results in differences in clinical presentation or response to treatment.

## Methods

### Ethical considerations

This study was approved by the Joint Clinical Research Centre (JCRC) Institutional Review Board (Kampala, Uganda) and the University Hospitals Cleveland Institutional Review Board (Cleveland, Ohio). Informed written consent was obtained from patients who participated in the study.

### Study population

Samples were collected from patients with at least one positive culture for *M. tuberculosis *who were previously enrolled in the Kawempe Community Household Contact Study, an ongoing epidemiological study in Kampala, Uganda, from which several papers have been published [15-17]. The Kawempe Community Household Contact study recruits patients who report not having received treatment for pulmonary tuberculosis.

Samples were collected consecutively from October 2007 to February 2009, from patients presenting with pulmonary TB symptoms who reported not having received treatment for TB in the preceding month. Demographic and clinical data, including age, sex, HIV status, and grade of chest radiograph were recorded.

### Samples and cultures

For each patient, sputum samples were collected at baseline and after two and five months of standard, short-course treatment. Auramine-stained sputum smears were examined for the presence of acid-fast bacilli. Samples were decontaminated and processed as described previously [18] using a standard sodium hydroxide-sodium citrate method. The processed samples were inoculated in Bactec-Mycobacterial Growth Indicator Tubes (MGIT) 960 culture tubes.

### Drug susceptibility testing

Drug susceptibility testing was performed on positive cultures at baseline and after five months of treatment.

### DNA extraction

Chromosomal DNA was extracted by centrifuging MTB cultures and re-suspending the cell pellet in Tris-EDTA buffer. Cells were then heat-killed at 95°C for 45 minutes, cooled to room temperature and centrifuged again. The supernatant containing chromosomal DNA was saved.

### Typing by MIRU-VNTR PCR

The MIRU-VNTR PCRs were performed on chromosomal DNA extracted from MTB cultures (described above). The PCR was designed to amplify a standard set of 15 MIRU-VNTR loci (580, 2996, 802, 960, 1644, 3192, 424, 577, 2165, 2401, 3690, 4156, 2164b, 1955 and 4052, see Table 1) from genomic DNA extracted from each sample using primers described by Supply et al [19] (Table 1). For each reaction, DNA from *M. tuberculosis *H37Rv was used as a positive control, and sterile water was used as a negative control. PCR products were electrophoretically separated on 2% agarose gels, using a 100-bp DNA ladder as size markers. From the gel images, the corresponding MIRU-VNTR bands were interpreted as copy numbers based on the reference table in the Supply 2005 protocol [20]. For any sample that showed multiple bands at any of the MIRU loci, the PCR was repeated at least twice in order to confirm the results. Multiple strains were noted to be present if a single sample had more than one PCR product at two or more loci, or if two or more samples from the same patient differed in copy numbers at two or more loci. Samples in which copy numbers were different at a single locus were considered to represent single strain evolution rather than multiple strains.

**Table 1:** PCR primer sequences and MIRU-VNTR locus designations1 used in this study

Locus	Alias	Repeat unit length, bp	PCR primer pairs (5'-3')
580	MIRU4, ETRD	77	GCGCGAGAGCCCGAACTGC GCGCAGCAGAAACGCCAGC

2996	MIRU26	51	TAGGTCTACCGTCGAAATCTGTGAC CATAGGCGACCAGGCGAATAG

802	MIRU40	54	GGGTTGCTGGATGACAACGTGT GGGTGATCTCGGCGAAATCAGATA

960	MIRU10	53	GTTCTTGACCAACTGCAGTCGTCC GCCACCTTGGTGATCAGCTACCT

1644	MIRU16	53	TCGGTGATCGGGTCCAGTCCAAGTA CCCGTCGTGCAGCCCTGGTAC

3192	MIRU31, ETRE	53	ACTGATTGGCTTCATACGGCTTTA GTGCCGACGTGGTCTTGAT

424	Mtub04	51	CTTGGCCGGCATCAAGCGCATTATT GGCAGCAGAGCCCGGGATTCTTC

577	ETR C	58	CGAGAGTGGCAGTGGCGGTTATCT AATGACTTGAACGCGCAAATTGTGA

2165	ETR A	75	AAATCGGTCCCATCACCTTCTTAT CGAAGCCTGGGGTGCCCGCGATTT

2401	Mtub30	58	CTTGAAGCCCCGGTCTCATCTGT ACTTGAACCCCCACGCCCATTAGTA

3690	Mtub39	58	CGGTGGAGGCGATGAACGTCTTC TAGAGCGGCACGGGGGAAAGCTTAG

4156	QUB-4156	59	TGACCACGGATTGCTCTAGT GCCGGCGTCCATGTT

2163b	QUB-11b	69	CGTAAGGGGGATGCGGGAAATAGG CGAAGTGAATGGTGGCAT

1955	Mtub21	57	AGATCCCAGTTGTCGTCGTC CAACATCGCCTGGTTCTGTA

4052	QUB-26	111	AACGCTCAGCTGTCGGAT CGGCCGTGCCGGCCAGGTCCTTCCCGAT

^1^: Extracted from Supply *et al*, [19]. Primers were synthesized by Eurofins MWG Operon, Ebersberg (Germany).

### Statistical analysis

Comparisons between the groups with multiple versus single strain infection were made using the student's t test for continuous variables, chi square for proportions, and multivariate regression analysis. A *p *value of < 0.05 was considered significant.

### Quality control

To avoid laboratory cross-contamination, cultures were done in small batches ensuring one sample is opened at a time. For each sample, separate tubes containing the digestion mixture and phosphate buffer were used to avoid cross-transfer of specimens. Sterile phosphate buffer was included in the batch being processed, and this remained negative when cultured. For each patient, multiple samples were cultured for a given time interval. A solitary positive sample at a given interval would trigger investigations for possible cross contamination.

For molecular assays, DNA extraction and PCR were conducted in a building and laboratory separate from the culture laboratory. The PCR laboratory has three separate rooms for Pre-amplification, amplification and post-amplification. Blank reactions (i.e. where no template DNA was added) were used to rule out cross contamination. A portion of all positive liquid cultures was used for ZN (Ziehl-Neelsen) staining, blood agar culture, and *IS6110*-PCR identification for MTB complex.

## Results

Between October 2007 and February 2009, samples were collected from a total of 113 patients with pulmonary tuberculosis. Eight of these patients were infected with multiple strains of *M. tuberculosis*, giving a prevalence of 7.1% (95% CI 2.35-11.81%) for multiple strain infections in our setting (see Table 2). Three patients who were infected with single strain were retreatment cases.

**Table 2:** Prevalence of multiple infections with M. tuberculosis and comparison between patients with multiple versus single strain infections

*Patients with pulmonary tuberculosis, N = 113*			
*Multiple strain infection, % (95% CI), 7.08% (2.35-11.81)*			
	**Multiple strains,** **n = 8**	**Single strain,** **n = 105**	***p *value**

**Baseline data**			

Age (median, years)	34.5	25	0.0745

Sex (% female)	50	51.89	0.8965

Body Mass Index (mean)	20.73	19.17	0.1764

HIV Positive (%)	37.5	12.62	0.0494

Sputum Smear Positive (%)	100	84.62	0.2169

Sputum Smear Grade (mean)	3.12	2.82	0.5511

Multidrug Resistance, n	0	1	0.8137

Culture positive after 2 months (%)	25.00	25.74	0.95

Culture positive after 5 months (%)	0	4.54	0.5379

			

**Chest X-ray^1^**			

Grade 0 (%)	25	8	0.1482

Grade 1 (%)	0	16	

Grade 2 (%)	0	25	

Grade 3 (%)	63	41	0.2813

			

**Co-morbidities**			

Respiratory tract infections (%)	88	53	0.0607

Malaria (%)	25	29	0.8289

Candidiasis (%)	25	6	0.0404

Anemia (%)	13	2	0.0723

PUD (%)	13	1	0.0170

			

**Drug resistance profiles**			

INH (%)	25	4	0.0573

RMP (%)	0	1	

EMB (%)	0	0	

PZA (%)	0	0	

STM (%)	0	2	

CI, Confidence Interval; **^1^**baseline chest x-ray data; PUD, Peptic ulcer disease; INH, Isoniazid; RMP, Rifampicin; EMB, Ethambutol; PZA, Pyrazinamide; STM, Streptomycin

At baseline, both patient groups (with multiple and single MTB strains) had similar proportion of females and body mass index (BMI). Though the multiple strain group had a higher median age, higher proportion with smear-positive tuberculosis, higher average sputum smear grade, and higher average grade of chest radiograph, these did not differ significantly between the two groups (Table 2). Three of the eight patients with multiple strain infections were HIV positive (37.5%), while 13 (12.62%) of those with single strain infections were HIV positive (p = 0.0494).

The underlying co-morbidities, as well as radiological findings and drug resistance profiles that were found in patients with single and multiple strain infections are summarized in Table 2. The number of patients with multiple strain infections was too small to make strong conclusions about these findings. Only one patient, who was infected with a single MTB strain, had multi-drug resistant tuberculosis.

Both groups had a similar proportion of patients with positive cultures after two months of treatment (multiple strains 25.00%, single strain 25.74%, p = 0.95). Four patients had positive cultures after five months, all of them infected with single strains (p = 0.5428).

Of the eight patients with multiple strain infections, seven had multiple strains in their baseline samples and only three had positive month two (M2) cultures. However, two of the M2 cultures were unrecoverable and the available M2 culture was confirmed with multiple strain infections. None of the eight patients had positive month five (M5) cultures (Table 3). Of the other seven patients, three had multiple strains in a single sputum sample, and four had different strains in different sputum samples.

**Table 3:** MIRU-VNTR genotypes of M. tuberculosis isolates from patients with multiple strain TB infections

			MIRU-VNTR Locus amplified														
***PTID***	***Time point***	***Sample***	**580**	**960**	**1644**	**2996**	**3192**	**802**	**424**	**577**	**1955**	**2163**	**2165**	**2401**	**3690**	**4052**	**4156**

A	BL	35357	2,5	2	4,5	3,4,6	1,3,5	3,4,6		4	2,6	1,3	4	1,2	3	2,6	5

	BL	35358	2,5	2	4,5	4	1,3	4		4	2	1		1		2	

																	

B	BL	33007	2	5	1,3,4	4,6	4	4,6,7	5	2		1,2,3	3	2,3,5		4	

																	

C	BL	34347	2	5	1	3	2	4		5	4	2	2	2		3	

	BL	34348	2	5	1	3	2,3	3,4,5		5,4,3	2,3,4	2	2,3,4	2,3,4		3	

																	

D	BL	33914	2	4	4	4	2	3	4	3	3	2	4	4	4	2	3

	BL	33913	2	4	4	3	1	2		4	3	2	4	2		2	3

																	

E	BL	35584	2	2	3	3	3	4	2	4	3	2	3	2	2	3	8

	BL	35583	3	4	5	3	2	3	2	4	2	2	4	2	4	3	3

																	

F	BL	37768	2	1	1	2	1	2		4	2	2	2	2	2	3	

	BL	37767	2	5	1	3	1	3	4	5	2	1	2	2	2	3	

																	

G	BL	38228	2	7	3	6,8	5	4		2	2,3,4	4	3	6		4	9

																	

H	BL	33027	MOTT														

	BL	33026	MOTT														

	M2	33608	2'	5	3	6	4	6	5	2		2	3	3		4	

	M2	33609	2	5	3	4,6			5	2,3		2	3	2, 3		4	

	M2	33610	2'	5	1	6		4	5	2		2	3	3		4	

The numerical figures (1 to 9) represent the number of alleles per amplified MIRU-VNTR loci, as described by Supply *et al *[19,20]. Where there is more than one numerical figure per locus implies existence of more than one MTB strain in the sample, indicating multiple strain infection with *M. tuberculosis *(Patient samples A, B, C, and G). Serial samples from patients D, E, F, and H had different strains at different sampling points, which also indicate multiple strains infection. For patients A-G, blanks in copy numbers are presumed to be due to technical laboratory error. For patient H, blanks are likely due to MOTT co-infection.

2' is a standard copy number noted in the MIRU manual; PTID, Patient Identification; BL, Baseline sample collection; M2, sample collection in the second month; MOTT, Mycobcateria Other Than Tuberculosis

## Discussion

This study demonstrates that infection with multiple strains of MTB occurs among patients with first episodes of pulmonary TB in a high-incidence community setting of Kampala. The prevalence of multiple strain infection in our cohort was 7.1%, the first MIRU-VNTR-based estimate from an East African country. In our study, the MIRU-VNTR loci were amplified using a single target ordinary PCR which, although proven sensitive, has a limit on detection and could have missed some infections with multiple MTB strains. Studies from similar high-incidence settings in South Africa showed multiple infection rates of 19%, in which PCR-based methods differentiated Beijing versus non-Beijing lineages, and 4.8% using spoligotyping [11], as well as 2.3% using IS*6110 *restriction fragment length polymorphism (RFLP) [9]. It is possible that the varying estimates are a reflection of differences in sensitivities of each method in detecting multiple MTB strains in sputum cultures, with PCR-based methods being more sensitive than RFLP. Presumably, a lower estimate of infections with multiple MTB strains would have been found had we used RFLP analysis in this cohort.

Baseline clinical presentations appear to be similar for patients with multiple and single strain infections. There was a higher rate of HIV infection in the multiple strain group, though this difference was not statistically significant using multivariate regression analysis. This study was limited by a relatively small sample size; further studies using a larger cohort would be useful in confirming or refuting these results. The average CD4 counts for patients with multiple versus single strain infection were similar, and patients with multiple strains had a wide range of baseline CD4 counts. If indeed HIV infection results in increased susceptibility to multiple strain infection, this vulnerability may apply at all CD4 counts, just as HIV-infected patients are more susceptible to TB disease at any CD4 count [21].

There was no difference in response to anti-tuberculosis treatment between the multiple and single infection groups; each group had similar rates of culture positivity after two and five months. There was a low rate of drug resistance in our cohort, similar to the overall rate in Uganda for multi-drug resistant TB (0.5% of new cases) [15]. Indeed, only one patient who was infected with a single MTB strain had MDR-TB.

The presence of infections with multiple MTB strains in this setting had little impact on the clinical course for individual patients. Multiple strain infections did not alter initial presentation or response to treatment. Additionally, drug resistance testing in our setting was not affected by the presence of multiple strains, but this seems to be mostly due to a low level of resistant strains in this community. In a setting with higher rates of drug resistance as well as a high level of TB transmission, it would seem likely that drug resistance testing can be complicated by multiple strain infections.

It is difficult to determine the timing of infection by individual strains resulting in multiple strain infections. Patients may be infected simultaneously by two or more strains, or an initial infection may be inadequate in providing immunity to subsequent re-infection with a new strain. It is also difficult to measure proportions of growth of different strains during the course of disease and which strains are primarily responsible for driving disease progression. Consequently, the results of molecular epidemiologic studies of TB transmission and recurrent disease should be interpreted in light of the possibility of multiple strain infection and varying transmission. When possible, such studies should employ methods sensitive enough to detect multiple infections. Additionally, the inability of initial infection to prevent subsequent infection adds to our understanding of host immunity and may influence development of an effective vaccine. The presence of multiple strains in 7.1% of TB patients in this setting also implies that there is a high rate of transmission and re-infection, emphasizing the importance of TB control in efforts to eradicate this disease.

One of the limitations in our study is that the MIRU-VNTR-PCR was done on genomic DNA extracts from cultured samples of MTB positive smears, and we could have missed MIRU-VNTR-positives from negative smear samples. However, doing direct PCR on sputum samples is associated with problems of sampling and inhibitors [22].

## Conclusion

Multiple strain infections occur among patients with first episodes of pulmonary tuberculosis in Kampala, in a setting with a high tuberculosis incidence. The presence of multiple strain infections had little impact on the clinical course for individual patients.

## List of abbreviations

MIRU-VNTR: Mycobacterial Interspersed Repetitive Unit-Variable-Number Tandem Repeats

## Competing interests

The authors declare that they have no competing interests.

## Authors' contributions

KRD designed and performed the baseline and laboratory procedures described, and drafted the initial manuscript; LN completed cultures and the MIRU-VNTR experiments; MLJ and DPK interpreted the data and critically revised the manuscript prior to submission; BBA, FAK, HMK and AO helped with data interpretation and sampling. The study was conceived by KRD, CW and MLJ.

All authors read and approved the final manuscript

## Pre-publication history

The pre-publication history for this paper can be accessed here:

http://www.biomedcentral.com/1471-2334/10/349/prepub
